# Novel Thogotovirus Associated with Febrile Illness and Death, United States, 2014

**DOI:** 10.3201/eid2105.150150

**Published:** 2015-05

**Authors:** Olga I. Kosoy, Amy J. Lambert, Dana J. Hawkinson, Daniel M. Pastula, Cynthia S. Goldsmith, D. Charles Hunt, J. Erin Staples

**Affiliations:** Centers for Disease Control and Prevention, Fort Collins, Colorado, USA (O.I. Kosoy, A.J. Lambert, D.M. Pastula, J.E. Staples);; University of Kansas Medical Center, Kansas City, Kansas, USA (D.J. Hawkinson);; Centers for Disease Control and Prevention, Atlanta, Georgia, USA (C.S. Goldsmith);; Kansas Department of Health and Environment, Topeka, Kansas, USA (D.C. Hunt)

**Keywords:** Thogotovirus, Bourbon virus, Heartland virus, viruses, high-throughput nucleotide sequencing, neutralization tests, febrile illness, death, Kansas, United States

## Abstract

A previously healthy man from eastern Kansas, USA, sought medical care in late spring because of a history of tick bite, fever, and fatigue. The patient had thrombocytopenia and leukopenia and was given doxycycline for a presumed tickborne illness. His condition did not improve. Multiorgan failure developed, and he died 11 days after illness onset from cardiopulmonary arrest. Molecular and serologic testing results for known tickborne pathogens were negative. However, testing of a specimen for antibodies against Heartland virus by using plaque reduction neutralization indicated the presence of another virus. Next-generation sequencing and phylogenetic analysis identified the virus as a novel member of the genus *Thogotovirus*.

## Abstract 2

Bourbon virus is a newly discovered pathogen associated with human illness and death.

The genus *Thogotovirus* (family *Orthomyxoviridae*) contains >6 distinct viruses, including Araguari, Aransas Bay, Dhori, Jos, Thogoto, and Upolu viruses (*1**–**3*). These viruses have been primarily associated with either hard or soft ticks and have a wide geographic distribution (*1**–**8*). The only virus in this genus known to occur in the United States is Aransas Bay virus, which was isolated from soft ticks (*Ornithodoros* spp.) collected from a seabird nest off the coast of Texas (*3*).

Two viruses in the genus *Thogotovirus* (Thogoto and Dhori viruses) are currently known to cause human infection and disease. Antibodies against Thogoto virus have been identified in humans living in parts of Europe, Asia, and Africa (*1**,**4**,**6**,**8*). Two persons from Nigeria infected with this virus were identified in 1966. The first patient was a man with a febrile illness in whom neuromyelitis optica later developed. The second patient was a 14-year-old boy in whom meningitis developed and who died 6 days later because of complications of sickle cell disease (*9*).

Antibodies against Dhori virus in humans have been reported in a similar distribution as those against Thogoto virus (*1**,**6**,**8**,**10*). Five patients with disease have been described after accidental laboratory exposure to Dhori virus; encephalitis developed in 2 of these patients (*11*). We report a novel Thogotovirus associated with a febrile illness and death that occurred in a man in the United States in 2014.

## The Case-Patient

The patient was a previously healthy man >50 years of age from Bourbon County, Kansas, USA. While working outdoors on his property in late spring 2014, the patient had several tick bites and found an engorged tick on his shoulder several days before he became ill with nausea, weakness, and diarrhea. The following day, a fever, anorexia, chills, headache, myalgia, and arthralgia developed. On the third day of illness, the patient went to his primary care physician, who empirically prescribed doxycycline for a presumed tickborne illness because of his history of tick bites, symptoms, and no reported travel outside the immediate area. The following morning, the patient’s wife found him obtunded (experiencing reduced consciousness) but arousable. He was taken by ambulance to a local hospital.

At the hospital, he had a temperature of 37.3°C, a pulse rate of 84 beats/min, and an increased blood pressure of 151/65 mm Hg. The patient had a papular rash on his trunk, but otherwise results of his physical examination were unremarkable. Initial laboratory findings showed leukopenia (2,200 cells/μL), lymphopenia (absolute lymphocyte count 550 cells/μL), thrombocytopenia (72,000 cells/μL), mild hyponatremia (sodium 133 mmol/L), hypokalemia (potassium 3.0 mmol/L), a creatinine level (0.8 mg/dL) within the reference range (0.6 mg/dL–1.2 mg/dL), a slightly increased level of blood urea nitrogen (25 μg/dL), and increased levels of aspartate aminotransferase (138 U/L) and alanine aminotransferase (86 U/L). He was admitted because of the principal problems of dehydration, syncope, and possible tickborne illness. He was given an intravenous (IV) fluid bolus, then maintenance fluids, and doxycycline (200 mg IV every 12 h for the first 24 h, then 100 mg IV every 12 h).

Despite doxycycline therapy, the patient continued to report malaise and anorexia, and periodic fevers (maximum temperature 38.8°C) developed. At day 8 postillness onset, the patient was transferred to a tertiary care center for further evaluation and management. Patient samples collected before transfer showed no serologic evidence of Rocky Mountain spotted fever, Lyme disease, or ehrlichiosis.

At initial assessment at the tertiary care center, the patient was febrile (temperature 39.4°C) and had a nontender left axillary lymphadenopathy; a diffuse maculopapular rash on his chest, abdomen, and back; petechiae on his soft palate and lower extremities; and bibasilar crackles in the lung fields. Laboratory testing continued to show mild leukopenia (3,600 cells/μL) but also showed worsening thrombocytopenia (34,000 cells/μL). His renal function was normal, but his aspartate aminotransferase level had increased to 119 U/L. Doxycycline treatment (100 mg IV every 12 h) was continued, and the patient was evaluated further for a potential etiology of his illness.

Hematologic results suggested that his persistent thrombocytopenia and leukopenia were secondary to acute bone marrow suppression. A chest, abdomen, and pelvis computed tomography scan with contrast showed trace pleural effusions, bibasilar atelectasis, and multiple prominent abdominal lymph nodes. At day 9 postillness onset, he remained lucid and interactive, but he continued to have episodic high fever (temperature >39°C) and progressive dyspnea developed, which resulted in a need for supplemental oxygen. A chest radiograph showed new findings of pulmonary venous congestion and interstitial edema, suggestive of progressive heart failure or fluid overload, and an echocardiogram showed global hypokinesis.

Because of increasing supplemental oxygen needs and progressive lactic acidosis, he was transferred to the intensive care unit and given broad-spectrum antimicrobial drugs on day 10 of his illness. His renal function began to deteriorate and his aminotransferase levels continued to increase. The patient was intubated because of acute respiratory distress syndrome and was given 3 vasopressor medications because of shock. The patient subsequently had sustained ventricular tachycardia with persistent hypotension and eventual pulseless electrical activity with refractory shock. After multiple resuscitations, the decision was made to withdraw further care, and he died shortly after being extubated, 11 days after first becoming ill. An autopsy was not performed.

Results of comprehensive evaluations for tickborne diseases, including serologic testing for Rocky Mountain spotted fever, tularemia, brucella, babesiosis, and Q fever; molecular testing for *Ehrlichia* spp. and *Anaplasma phagocytophilum*; and blood thin smears for *Babesia* spp. were negative. Results of evaluations for fungal pathogens (*Aspergillus* spp. galactomannan, antibodies against *Histoplasma* spp., and *Histoplasma* spp. antigen in serum and urine) were negative. Evaluations for cytomegalovirus, Epstein-Barr virus, and parvovirus showed past infection. Test results for hepatitis B and C viruses, West Nile virus, and HIV were also negative. Blood, sputum, and urine bacterial cultures were negative. A whole blood specimen collected 9 days after illness onset was sent to the Centers for Disease Control and Prevention (CDC) (Fort Collins, CO, USA) for Heartland virus testing as part of an active institutional review board-approved protocol.

## Materials and Methods

### Clinical Specimen Handling and Evaluation

At CDC, EDTA-treated blood, along with serum separated from that blood, were tested for Heartland viral RNA and neutralizing antibodies by real-time reverse transcription PCR (RT-PCR) and plaque reduction neutralization test (PRNT) with 6-well plates with confluent Vero E6 monolayers, according to protocols described elsewhere (*12**,**13*). Standard virus isolation methods were also used. In brief, 200 μL of undiluted and 1:10 dilutions of blood or serum specimens were inoculated onto confluent Vero cells in T25 flasks. Inoculated flasks were then incubated at 37°C and reviewed for cytopathic effect daily.

### Viral Genome Sequencing, RT-PCR, and Phylogenetic Analyses

Supernatants collected from standard virus isolation cell cultures were subjected to next-generation sequencing (NGS) methods by using the Ion Torrent PGM sequencer (Life Technologies, Grand Island, NY, USA) and methods as described (*14*). After novel viral sequences were identified by NGS, a real-time RT-PCR was designed to target the newly derived sequences and applied to blood and serum samples by using methods described (*15*). Phylogenetic analyses were conducted on deduced amino acid sequences from multiple genomic segments of selected viruses of the same viral family by using MEGA 5.05 software (http://www.megasoftware.net/) as described (*16*).

## Results

### Isolation and Identification of Virus in Blood and Serum

**Figure 1:**
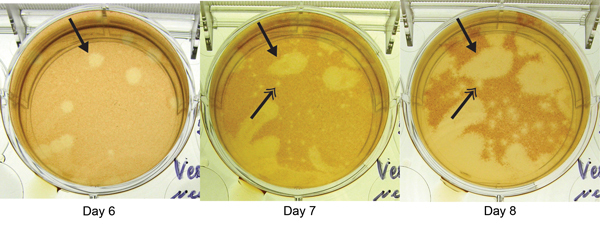
Plaque reduction neutralization test of patient sample for Heartland virus, showing images of the same well obtained days 6, 7, and 8 postinoculation at a dilution of 1:20. Arrows with single heads indicate appearance of a novel virus plaque beginning at day 6. Arrows with double heads indicate development of a typical Heartland virus plaque, apparent on day 7 and more evident on day 8, generated from a control strain added to each well in defined quantities to identify Heartland virus–specific antibodies in the patient sample.

Blood and serum showed negative results for Heartland viral RNA and antibodies against this virus. However, heterologous viral (non–Heartland viral) plaques were noted in PRNT cell culture wells, which indicated the presence of another virus (Figure 1). Standard virus isolation methods showed a substantial cytopathic effect at day 3 postinoculation in cells that were inoculated with blood or serum specimens. These findings were confirmed by repeated isolation attempts.

**Figure 2:**
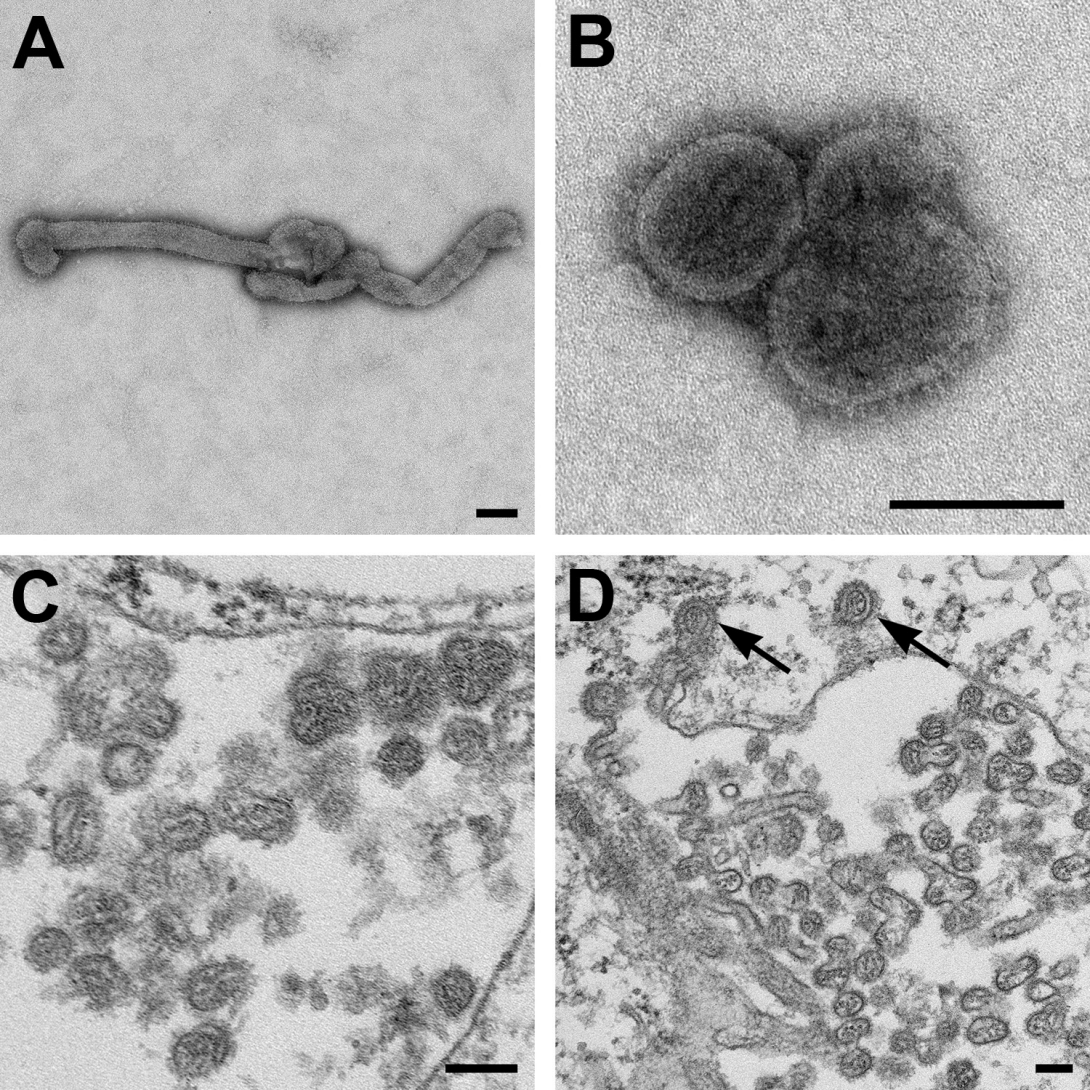
Electron microscopic images of novel Thogotovirus isolate. Filamentous (A) and spherical (B) virus particles with distinct surface projection are visible in culture supernatant that was fixed in 2.5% paraformaldehyde. Thin-section specimens (C and D), fixed in 2.5% glutaraldehyde, show numerous extracellular virions with slices through strands of viral nucleocapsids. Arrows indicate virus particles that have been endocytosed. Scale bars indicate 100 nm.

Negative stain and thin-section electron microscopy showed pleomorphic viral particles consistent with viruses in the family *Orthomyxoviridae* (Figure 2). NGS methods applied to cell culture supernatants from multiple isolations showed the presence of novel orthomyxoviral RNA. We observed ≈70% overall average nucleotide sequence percentage identity with Dhori virus in multiple genomic segments. Blood and serum samples were verified as the source of the novel virus by real-time RT-PCR–based detection of viral RNA in these samples.

### Phylogenetic Analyses

**Figure 3:**
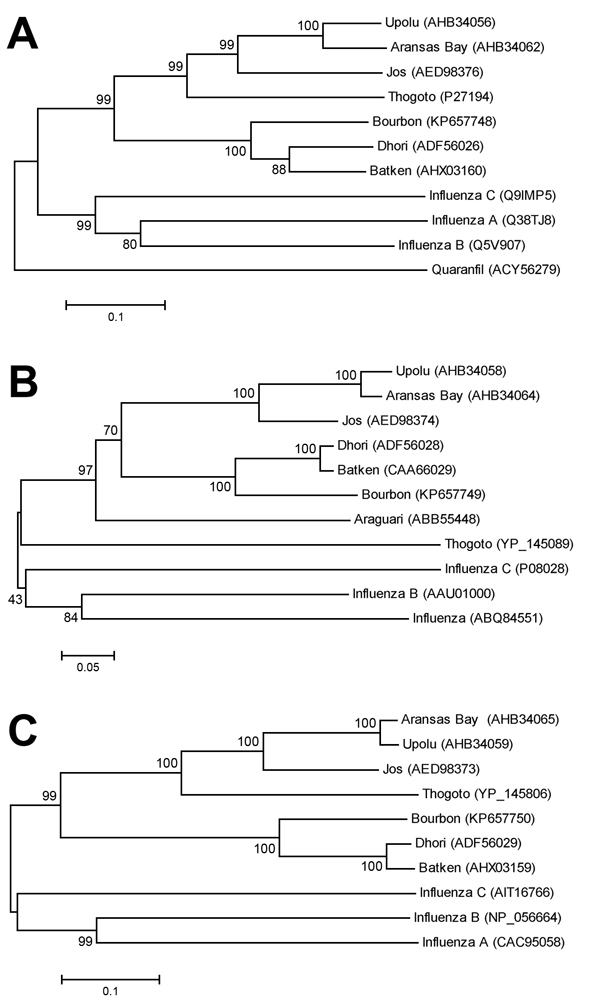
Phylogenies of deduced amino acid sequences of representative genes of Bourbon virus in comparison to homologous sequences of selected orthomyxoviruses. A neighbor-joining method was used for inference of each phylogeny with 2,000 replicates for bootstrap testing. Values at nodes are bootstrap values. A) PA polymerase subunit (segment 3). B) Nucleocapsid protein (segment 5). C) Membrane protein (segment 6). GenBank accession numbers appear next to taxon names. Scale bars indicate number of amino acid substitutions per site.

Three phylogenies, each generated by a neighbor-joining method applied with 2,000 bootstrap replicates for grouping analysis, were chosen as representative of overall genetic relationships of selected viruses (Figure 3). The novel virus was found to group with strong support along with Dhori virus, and the closely related Batken virus, in all trees.

## Discussion

Using traditional techniques (i.e., PRNT and culturing on animal cells) in combination with NGS, we isolated a novel virus from a blood sample collected 9 days after illness onset from a previously healthy man. It is likely that this novel Thogotovirus, which we are proposing to call Bourbon virus after the county of residence of the patient, was the cause of his illness. Although it is unclear what role the virus played in the death of the patient, the high level of viremia, as shown by multiple isolations from the blood of the patient 2 days before his death, suggests that this might have contributed to the death of the patient.

The patient had a history of tick exposure, as well as symptoms and laboratory findings (i.e., leukopenia and thrombocytopenia) consistent with a tickborne illness. Several tickborne pathogens, such as *Ehrlichia chaffensis*, *Rickettsia*, and Heartland virus, are present in eastern Kansas and adjacent areas (*17**–**19*). However, the patient did not respond to doxycycline therapy initiated 3 days after illness onset and had negative results for these and other tickborne pathogens.

Of the 7 symptomatic human infections that have been associated with viruses in the genus *Thogotovirus*, most case-patients have had neurologic findings (e.g., meningitis, encephalitis) without any described abnormalities in blood counts (*9**,**11*). Although cerebrospinal fluid was not tested for the patient reported, his clinical signs and symptoms were not suggestive of neurologic infection. Furthermore, the patient did not have any respiratory symptoms that would be expected with other viruses that are known human pathogens in the large family of *Orthomyxoviridae*, such as influenza virus (*1*).

Phylogenetic analyses indicated that Bourbon virus is most closely related to Dhori and Batken viruses. However, the branch lengths suggest a relatively distant evolutionary distinction of Bourbon virus from Dhori and Batken viruses, which have only been described in the Eastern Hemisphere. Dhori, Batken, and Thogoto viruses have been identified in various hard tick species (*1*). However, Batken virus also has been identified in mosquitoes (*1*). It is currently unknown how Bourbon virus is transmitted to humans. However, illness onset of the patient in late spring and a history of finding an embedded tick before becoming ill support the notion that Bourbon virus might be transmitted by ticks. Therefore, to potentially prevent Bourbon virus disease, as well as other tickborne diseases, persons should be advised to avoid tick bites by using an insect repellent registered with the US Environmental Protection Agency to be effective against ticks, wearing long sleeves and pants, avoiding bushy and wooded areas, and performing tick checks after spending time outdoors.

The discovery of Bourbon virus, in addition to recent discoveries of tick-associated Heartland and severe fever with thrombocytopenia syndrome viruses (*19**,**20*), suggests that the public health burden of these pathogens has been underestimated. As nonselective molecular methods of pathogen identification (i.e., NGS sequencing) become more widely used, ideally in combination with classical microbiologic techniques, it is anticipated that similar discoveries will be made in the future.

It is currently not known how many human infections and disease cases might be attributable to this novel pathogen. On the basis of limited information for our case-patient, health care providers might consider Bourbon virus as a potential infectious etiology in patients in whom fever, leukopenia, and thrombocytopenia develop without a more likely explanation and who have shown negative results for other tickborne diseases (e.g., ehrlichiosis, anaplasmosis, or Heartland virus disease) or have not responded to doxycycline therapy. Work is planned to identify additional human infections with this novel virus, as well as to explore its potential geographic distribution. Also, more comprehensive virologic characterizations and field work are ongoing to better understand the biology of, and to identify potential vectors and reservoirs for, Bourbon virus. These data will be critical to further characterize the epidemiology and illness caused by Bourbon virus and to implement potential prevention and control measures.
